# Prognosis of Elderly Japanese Patients Aged ≥80 Years Undergoing Hemodialysis

**DOI:** 10.1155/2013/693514

**Published:** 2013-10-09

**Authors:** Shingo Hatakeyama, Hiromi Murasawa, Itsuto Hamano, Ayumu Kusaka, Takuma Narita, Masaaki Oikawa, Daisuke Noro, Kazuhisa Hagiwara, Hirofumi Ishimura, Takahiro Yoneyama, Yasuhiro Hashimoto, Takuya Koie, Hisao Saitoh, Tomihisa Funyu, Chikara Ohyama

**Affiliations:** ^1^Department of Urology, Hirosaki University Graduate School of Medicine, 5 Zaifu-chou, Hirosaki 036-8562, Japan; ^2^Department of Urology, Oyokyo Kidney Research Institute, Hirosaki 036-8243, Japan; ^3^Department of Advanced Transplant and Regenerative Medicine, Hirosaki University Graduate School of Medicine, Hirosaki 036-8562, Japan

## Abstract

Although the number of elderly patients requiring dialysis has increased, data regarding the prognosis of elderly patients undergoing hemodialysis are limited. In the present study, prognosis in Japanese hemodialysis patients aged ≥80 years was evaluated. From January 1988 to July 2013, 1144 consecutive patients with end-stage renal disease required renal replacement therapy at our institution; of these, 141 were aged ≥80 years. These patients' charts were retrospectively reviewed for relevant clinical variables and survival time. The life expectancies table from the National Vital Statistics database was used, and prognostic factors were assessed by multivariate analysis. In total, 107 deaths (76%) were recorded during the study period. The median survival time and estimated life-shortening period in the patients were 2.6 years and −5.3 years, respectively. Eastern Cooperative Oncology Group Performance Status and hemoglobin level were revealed as prognostic factors in the multivariate analysis. Estimates of prognosis and prognostic factors may provide useful information for physicians as well as elderly patients with end-stage kidney disease.

## 1. Introduction

**Figure 1:**
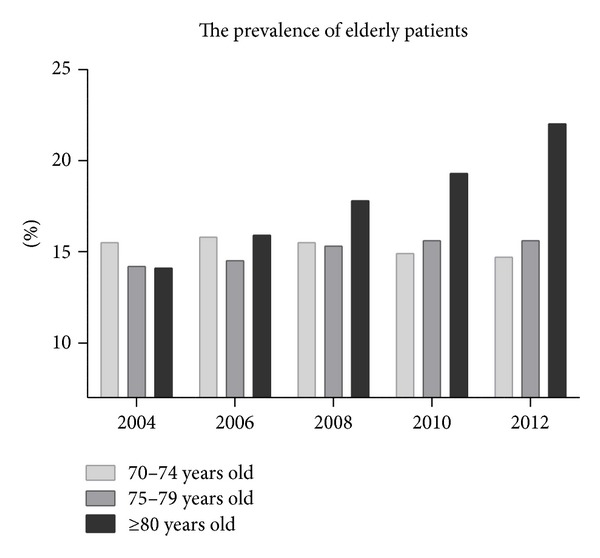
Prevalence of elderly patients receiving dialysis treatment in the overall Japanese population according to age.

As the Japanese population continues to age and the prevalence of chronic kidney disease increases [1, 2], clinicians are frequently faced with the decision of whether or not to initiate renal replacement therapy for their patients. According to the latest nationwide review conducted by the Japanese Society for Dialysis Therapy in 2012, 309,946 patients were on dialysis, and dialysis was initiated in 38,165 new patients that year [3]. Along with this increase in the number of dialysis patients, the number of older patients (≥80 years) undergoing hemodialysis treatment each year has also increased. In 2004, 14% of all dialysis patients in Japan were ≥80 years old. These figures were 16% in 2006, 18% in 2008, 19% in 2010, and 22% in 2012, whereas the number of Japanese patients aged 70–79 years receiving dialysis has remained unchanged in the last decade (Figure 1) [3].

Many clinicians believe that age is a barrier for initiation of renal replacement therapy because dialysis in elderly patients has been associated with an increased risk of mortality. However, data regarding the prognosis of elderly patients undergoing hemodialysis are limited. Thus, in the present study, the median survival time in hemodialysis patients aged ≥80 years was evaluated, and the period of time by which these patients' lives were shortened (life-shortening period) was estimated using a life expectancies table from the National Vital Statistics data for 2008 [4]. Prognostic factors were then assessed by multivariate analysis.

## 2. Materials and Methods

This study was conducted in accordance with the ethical standards of the Declaration of Helsinki and approved by the Institutional Ethics Committee. From January 1988 to July 2013, 1144 consecutive patients with end-stage renal disease required renal replacement therapy at the Oyokyo Kidney Research Institute, Hirosaki, Japan. Of these, 141 were aged ≥80 years. Patient charts were retrospectively reviewed for relevant clinical variables and survival time.

The following data were collected for use in the analyses: patient age, gender, body mass index, and blood pressure; hemoglobin, serum albumin, phosphorus, potassium, and corrected calcium levels; blood urea nitrogen level and estimated glomerular filtration rate (eGFR); concomitant use of antihypertensive drugs (angiotensin-converting enzyme inhibitors, angiotensin receptor blockers, or calcium blockers); and presence or absence of diabetes mellitus or cerebral and cardiovascular disease (cerebral infarction, heart failure, myocardial infarction, and angina pectoris) at the initial visit. The eGFR was calculated using values for age, gender, and serum creatinine levels and the equation shown below [5]. This eGFR equation for Japanese patients is a modified version of the abbreviated Modification of Diet in Renal Disease Study formula: eGFR mL/min/1.73 m^2^ = 194 × sCr^−1.094^ × age^−0.287^ (×0.739, if female) [6]. Patient general health status before dialysis initiation was evaluated on the Eastern Cooperative Oncology Group Performance Status scale (ECOG-PS) [7].

The life expectancy is calibrated using the life expectancies table [4] based on expected age of death on specific age at dialysis initiation. To evaluate differences in life expectancy between these patients and the general population, life-shortening periods were calculated according to the following formula: expected age of death on specific age at dialysis initiation—the actual age of death. 

### 2.1. Basic Policies for Indication of Renal Replacement Therapy

Hemodialysis is the standard treatment strategy for renal replacement therapy in elderly patients (≥80 years) with end-stage renal disease at our institution. The purpose of this treatment is to minimize present suffering, gain time to consider continuation of renal replacement therapy and its alternatives, and ensure renal survival. Patients who refuse renal replacement therapy and those with systemic comorbidities, extremely advanced heart failure, or severe complications are designated as “not indicated for treatment.”

### 2.2. Follow-Up Schedule

All patients were routinely followed up for thrice-weekly hemodialysis with standard care according to the guidelines of the Japanese Society for Dialysis Therapy for the management of patients on chronic hemodialysis [8, 9] and tracked until the occurrence of death, loss of follow-up, or end of study (July 31, 2013), whichever came first. Erythropoiesis-stimulating agents were used when hemoglobin level was lower than 10 g/L in all patients. The target hemoglobin level was 10-11 g/L. 

### 2.3. Statistical Analysis

Patient survival was evaluated using the Kaplan-Meier method. Variables were compared among groups using Student's *t*-test or Mann-Whitney *U* test. Age, gender, body mass index, blood pressure, hemoglobin, serum albumin, phosphorus, potassium, and corrected calcium levels, blood urea nitrogen, estimated glomerular filtration rate (eGFR), concomitant use of antihypertensive drugs, presence or absence of diabetes mellitus, and cerebral and cardiovascular disease were analyzed using stepwise Cox regression multivariate analysis to determine independent predictors for overall survival. After these factors were identified, a receiver operating characteristic (ROC) curve was used to determine the optimal cut-off value for prognosis. This value was calculated using the following formula [10]: (1 − sensitivity)^2^ + (1 − specificity)^2^. Each patient was categorized according to the number of risk factors identified in the Cox regression multivariate analysis to evaluate the predictive potential of risk criteria for prognosis. Each positively identified risk factor was given a score of 1, and scores for all other risk factors were summed. Patients were classified into three groups according to the number of risk factors: the low-risk group (patients with no risk factors), the intermediate-risk group (one risk factor), and the high-risk group (two risk factors). The statistical significance of the differences between the three groups was evaluated by the log rank test.

All statistical analyses were performed using the SPSS software package version 19.0 (SPSS, Chicago, IL, USA) and GraphPad Prism version 5.03 (GraphPad Software, San Diego, CA, USA). A *P* value of <0.05 was considered statistically significant.

## 3. Results

**Figure 2:**
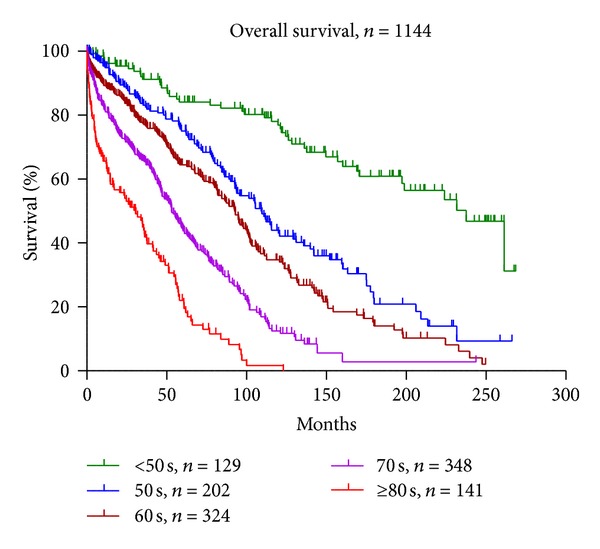
Overall survival in 1144 dialysis patients according to age.

Characteristics of all 1144 dialysis patients are summarized in Table 1. The age distribution of patients was as follows: 129 (11%), 202 (18%), 324 (28%), 348 (30%), and 141 (12%) in the following age groups: <50 years (<50 s), 50–59 years (50 s), 60–69 years (60 s), 70–79 years (70 s), and ≥80 years (≥80 s), respectively. The median survival times were 20, 9.1, 7.7, 4.4, and 2.6 years in the same age groups (Figure 2). The most frequent and second most frequent causes of death were cerebro-cardiovascular disease and infectious diseases, respectively, except for patients in the ≥80s group. Mortality increased with age. The estimated mean life-shortening values were −30.1, −22.5, −14.6, −9.4, and −5.3 years, respectively, in deceased patients in the five age groups.

**Figure 3:**
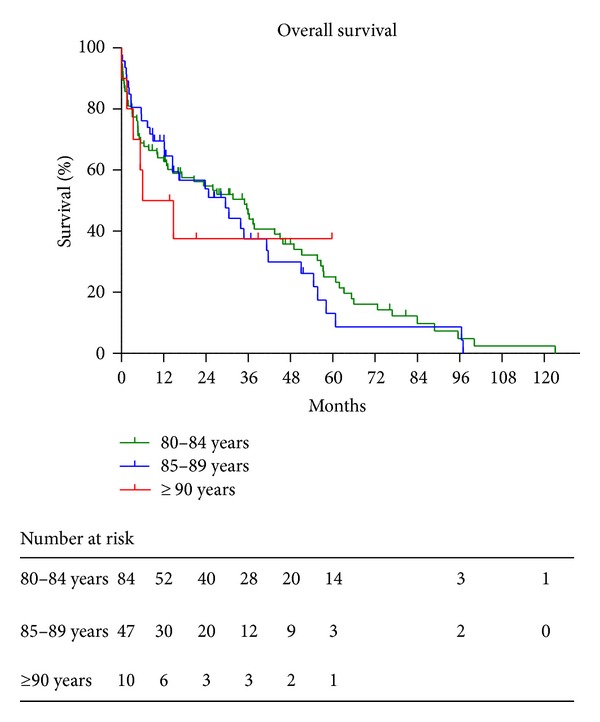
Overall survival in 144 dialysis patients aged ≥80 years. The median survivals in the age groups of 80–84, 85–89, and >90 years were 3.0, 2.5, and 0.9 years, respectively. There were no significant differences in survival among the groups.

**Figure 4:**
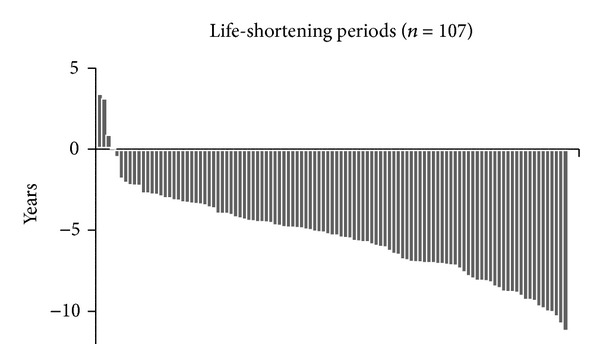
Life-shortening periods in dialysis patients aged ≥80 years. The median estimated life-shortening periods were −5.3 years in deceased patients aged ≥80 years.

Characteristics of the 141 patients in the ≥80s group are summarized in Table 2. All patients were undergoing hemodialysis, and their median age was 83 years. The median survival times in patients aged 80–84, 85–89, and >90 years old were 3.0, 2.5, and 0.9 years, respectively (Figure 3). Among the 141 patients, 107 deaths (76%) were recorded as of July 31, 2013. The highest number of patients who died within 1 year was 49 (46%). The most frequent causes of death were infectious disease in 35 patients (33%), and the second most frequent causes of death were cerebro-cardiovascular disease in 29 patients (27%). The median estimated life-shortening period was −5.3 years in deceased patients aged ≥80 years. Only three patients survived longer than the general life expectancy in the Japanese population (Figure 4). 

**Figure 5:**
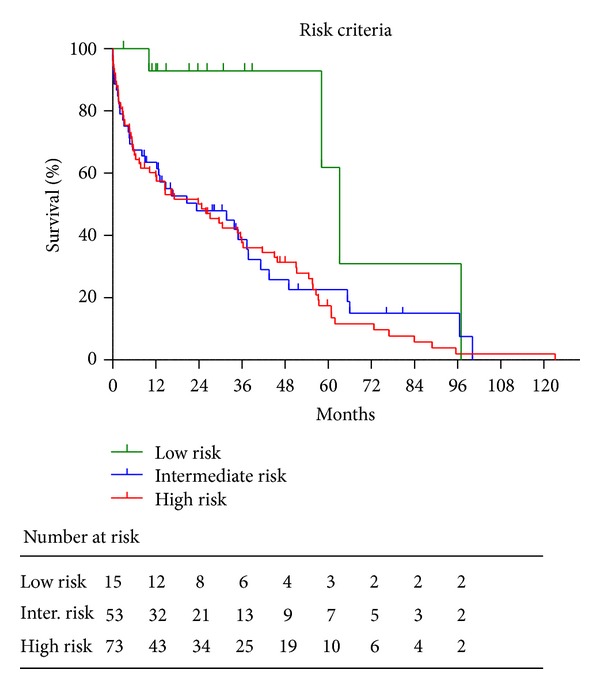
Risk classification for prognosis in dialysis patients aged ≥80 years. Each patient was categorized according to the number of selected risk factors using Cox regression multivariate analysis to evaluate the predictive potential of risk criteria for prognosis. Each existing risk factor was scored as 1, and scores for all the other risk factors were summated. Patients were classified into three groups according to the number of risk factors: the low-risk group (patients with no risk factor), the intermediate-risk group (1 risk factors), and the high-risk group (2 risk factors).

Results of the multivariate analysis revealed ECOG-PS and hemoglobin levels as significant prognostic factors in elderly patients undergoing hemodialysis (Table 3). Risk criteria were constructed using these significant independent risk factors for stratification of patient survival. The optimal cut-off points calculated from ROC curves for ECOG-PS and hemoglobin level were >1 and <9.55 g/L, respectively. Patients were then categorized according to the number of independent risk factors for overall survival. This risk classification indicated significantly poor prognoses in the intermediate- and high-risk groups compared with those in the low-risk group (*P* = 0.0059) (Figure 5). The median survival time in the low-risk group was 63 months, whereas that in the other groups was 23-24 months. 

## 4. Discussion

In Japan, the number of elderly patients with end-stage renal disease requiring dialysis treatment continues to grow. Data from the latest Japanese Society for Dialysis Therapy database (2012) showed that the number of dialysis patients aged ≥80 years increased by more than 8% between 2005 and 2013 [3], and 22% of all dialysis patients were aged ≥80 years. Several studies have particularly evaluated the indications and outcomes of maintenance dialysis in elderly patients [11–18]; however, no reports have examined prognosis in these patients in Japan. In this study, survival outcome in elderly Japanese hemodialysis patients aged ≥80 years was compared with that in the general population. The median survival time of 2.6 years was comparable to that in previous reports of approximately 2.4–3.2 years [13, 16, 17, 19].

Japan is a country where life expectancy is high [4, 20]. Thus, in the general population, life expectancy among patients aged ≥80 years is 7.6 years. The estimated median life-shortening period calculated from the life expectancies table was −5.3 years in deceased patients. These data could not be compared with those from other industrialized countries; however, comparison using the estimated median life-shortening periods from various countries may reveal social differences, including those related to medical or insurance systems. Further studies are required on this issue.

Because elderly patients on hemodialysis constitute a heterogeneous group of patients, their chronological age may not necessarily correlate with their biological age. Several risk factors pertain to elderly hemodialysis patients aged ≥80 years, including body mass index, late referral to a nephrologist, poor performance status, presence of peripheral vascular disease [17], older age, acute congestive heart failure, any walking impairment, and hemoglobin level (<10 g/L) [18]. Establishing a standard risk-associated classification system for planning dialysis initiation may help clinicians in treatment decision-making. In the multivariate Cox analysis, independent predictors for overall survival were ECOG-PS ≥1 and hemoglobin level was ≤9.55 g/L. Based on these risk criteria, survival rates were significantly lower in the intermediate- and high-risk groups.

Because of technological advancements in dialysis treatment, old age is no longer considered as a contraindication in most industrialized countries [15–18, 21, 22]. Recent studies suggested that dialysis provides a survival benefit compared with conservative management for patients with stages 4-5 chronic kidney disease over the age of 75 years [23–25]. However, dialysis may or may not offer a substantial prolongation of life expectancy with an acceptable quality of life (QOL) among elderly patients. QOL is very important for older patients for whom renal transplantation is an unlikely option. However, very few studies have addressed QOL issues in elderly patients with end-stage renal disease because of the controversial nature of this decision [19, 21, 26]. Lamping et al. reported no significant differences in QOL scores between elderly dialysis patients and elderly individuals in the general population in the UK and USA [26]. In contrast, Tamura et al. reported an association between dialysis initiation and substantial and sustained decline in functional status among nursing home residents with end-stage renal disease [27]. Carson et al. demonstrated prolonged survival for elderly patients (≥70 years of age) on dialysis compared with those conservatively treated. However, survival time in patients who were conservatively treated may be substantial, considering that a similar number of hospital-free days was recorded for both groups of patients [19]. Benefits and disadvantages of dialysis in elderly patients and its effects on QOL continue to be debated. The particular needs and traditional customs of the populations in each individual country or region must be considered. In addition, because randomized controlled trials comparing outcomes and QOL in patients receiving renal replacement therapy compared with those conservatively treated are not feasible, observational studies remain the only means by which treatment methods can be compared.

The present study has several important limitations, including its retrospective nature, small sample size, and the inclusion of patients within a single institution. We could not address the total dose and its influences of erythropoiesis-stimulating agents. There might be association between the dose of erythropoiesis-stimulating agents and patients' death. Higher use of erythropoiesis-stimulating agents for poor responder or excessively higher hemoglobin levels may link to poor prognosis. Therefore, the results of this study cannot be generalized. In addition, the primary question as to who will receive survival and comprehensive benefits may remain unanswered. In elderly patients undergoing hemodialysis, details of patient characteristics such as late referral, presence of peripheral vascular disease, frailty, and information regarding QOL were lacking and must therefore be considered in future studies.

Despite these limitations, the present study provided some useful information. Shortening of life expectancy was investigated using the National Vital Statistics survey database for Japan in 2008. Because medical and insurance systems differ among countries, decision-making regarding dialysis initiation and its indications may also differ. Because Japan is a country with a high life expectancy rate [4, 20], life expectancy in elderly patients should be calculated using life expectancy tables in each individual country or region. The present study is the first to investigate the shortening of life expectancy in Japanese hemodialysis patients aged ≥80 years. 

## 5. Conclusion

In conclusion, the results of this study shed light on the prognosis of elderly dialysis patients in Japan, whose number is increasing. Our observations suggest that old age is no longer considered as a contraindication, and hemodialysis initiation is acceptable for the rather elderly patients with end-stage renal disease in consideration of risk factors. Further clinical study is required to determine the most appropriate treatment for elderly patients with end-stage renal failure. A new evaluation system is required to aid decision-making between conservative or renal replacement therapy with consideration of comorbidities, health status, and patient preferences in the elderly Japanese population.

## Figures and Tables

**Table 1:** Characteristics of 1144 dialysis patients included in this study.

	All	<50 years	50s	60s	70s	≥80s
Number of patients	1144	129	202	324	348	141
Male/female	714/430	78/51	137/65	209/115	217/131	73/68
Age at start dialysis (years)	65.8 ± 12.9	41.1 ± 7.2	55.8 ± 2.8	64.7 ± 2.9	74.5 ± 3.1	84.2 ± 3.1
Survival after dialysis initiation (years)	6.8	20	9.1	7.7	4.4	2.6
Life expectancy (years)	18.6	40.2	28.1	20.2	13.4	7.7
Life-shortening periods (years)	−11.6	−30.1	−22.5	−14.6	−9.4	−5.3
Deceased patients	667 (58%)	39 (30%)	108 (53%)	182 (56%)	230 (66%)	107 (76%)
Cause of death						
Cerebro-cardiovascular disease	235 (35%)	14 (36%)	41 (38%)	75 (41%)	76 (33%)	29 (27%)
Infections	167 (25%)	7 (18%)	27 (25%)	37 (20%)	61 (27%)	35 (33%)
Cancer	88 (13%)	5 (13%)	14 (13%)	25 (14%)	30 (13%)	14 (13%)
Others	132 (20%)	7 (18%)	17 (16%)	30 (16%)	22 (10%)	22 (21%)
Unknown	44 (7%)	6 (15%)	9 (8%)	15 (8%)	7 (3%)	7 (6.5%)

**Table 2:** Characteristics of dialysis patients aged ≥80 years.

	All	Living	Deceased	*P* value
Number of patients	141	34	107	
Male/female	73/68	17/17	56/15	*0.0026 *
Duration from first visit to dialysis initiation (months)	4.2 ± 13	8.3 ± 18	4.2 ± 11	*0.2154 *
Age at start dialysis (years)	84 ± 3.1	84.5 ± 3.6	84.1 ± 3.0	*0.5540 *
Deceased within 1 year			49 (46%)	
ECOG-PS at dialysis initiation	2.2 ± 1.3	1.2 ± 1.4	2.2 ± 1.2	*0.0004 *
Cerebro-cardiovascular disease	88 (62%)	20 (59%)	68 (64%)	*0.6200 *
Diabetes mellitus	49 (35%)	11 (32%)	38 (36%)	*0.7360 *
Mean blood pressure (mmHg)	117 ± 21	114 ± 22	117 ± 22	*0.4817 *
Systolic	156 ± 31.7	159 ± 34.1	156 ± 31.1	*0.7437 *
Diastolic	78 ± 16	69.6 ± 15.6	77.6 ± 16.2	*0.0132 *
Body mass index	22 ± 4.3	23.2 ± 4.0	22.2 ± 4.4	*0.2319 *
Hemoglobin (g/L)	8.9 ± 1.9	9.2 ± 1.9	8.9 ± 1.9	*0.4054 *
BUN (mg/dL)	84 ± 33	76.7 ± 33.7	83.5 ± 33.1	*0.3071 *
eGFR	8.4 ± 4.5	7.9 ± 4.1	8.4 ± 4.7	*0.5604 *
Albumin (mg/dL)	3.4 ± 0.6	3.5 ± 0.5	3.4 ± 0.7	*0.1813 *
Phosphorus (mg/dL)	5.3 ± 1.6	5.0 ± 1.3	5.3 ± 1.7	*0.2801 *
Potassium (mEq/L)	4.8 ± 1.0	5.0 ± 0.8	4.8 ± 1.0	*0.3065 *
Calcium (mg/dL)	8.8 ± 0.7	8.4 ± 0.6	8.1 ± 0.8	*0.0110 *

**Table 3:** Results of univariate and multivariate Cox regression analyses for overall survival in dialysis patients aged ≥80 years.

	Univariate analysis				Multivariate analysis		
	*P* value	HR	95.0% CI		*P* value	HR	95.0% CI
Age (years)	*0.443 *	1.024	0.96–1.09				
Cerebro-cardiovascular disease	*0.163 *	1.327	0.89–1.98				
Diabetes mellitus	*0.976 *	0.994	0.67–1.46				
ECOG-PS	*0.000 *	1.327	1.15–1.54	ECOG-PS	*0.004 *	1.27	1.08–1.49
Mean blood pressure (mmHg)	*0.164 *	0.994	0.99–1.01				
Body mass index	*0.060 *	0.961	0.92–1.01				
Hemoglobin (g/L)	*0.231 *	1.070	0.96–1.20	Hemoglobin	*0.040 *	1.13	1.01–1.28
BUN (mg/dL)	*0.177 *	1.004	0.99–1.01				
eGFR (mL/min./1.73 m^2^)	*0.782 *	1.006	0.97–1.05				
Albumin (mg/dL)	*0.013 *	0.652	0.47–0.91	Albumin	*0.067 *	0.71	0.48–1.03
Phosphorus (mg/dL)	*0.068 *	1.132	0.99–1.29				
Potassium (mEq/L)	*0.265 *	1.133	0.91–1.41				
Calcium (mg/dL)	*0.861 *	1.024	0.79–1.33				
Antihypertensive agents	*0.275 *	1.324	0.80–2.19				
